# WE BUILT IT, BUT THEY DIDN’T COME: CHALLENGES WITH RECRUITMENT AND RETENTION IN THE CONFIDENCE PILOT STUDY

**DOI:** 10.1093/geroni/igad104.2001

**Published:** 2023-12-21

**Authors:** Alexander Gonzalez, Shane Rhodes, Donna Benton, Kylie Meyer

**Affiliations:** University of Southern California, Los Angeles, California, United States; University of Texas Health Science Center at San Antonio, San Antonio, Texas, United States; University of Southern California, Los Angeles, California, United States; Case Western Reserve University, Cleveland, Ohio, United States

## Abstract

This presentation considers reasons for low attendance in a psychoeducational intervention developed to reduce financial strain and manage out-of-pocket caregiving costs. From May 2022 until February 2023, the CONFIDENCE Financial Education Intervention was delivered to six cohorts of family caregivers to persons living with dementia. Delivery of the 5-week, group-based Zoom intervention occurred at an academic service center in Los Angeles, CA that regularly provides caregiver education. Whereas the program team anticipated 192 caregivers to attend over 10 months, 72 caregivers registered in this time, among whom just 38 attended at least one session. The average number of sessions attended was 3.0 (SD=1.5) of five. To understand possible reasons for low participation, the investigators conducted bivariate comparisons of the demographic characteristics of attendees versus non-attendees from registration forms and reviewed fidelity reports. Fidelity reports were completed using a standard form using Likert-scale responses ranging from 0 (Strongly Disagree) to 5 (Strongly Agree). Forms were completed by facilitators after delivering sessions and by an independent observer who reviewed a lesson recording. Evaluation of registration forms showed no statistically significant difference among caregivers who attended at least one session versus those who did not attend according to caregiver age, ethnicity, race, or income. Further, fidelity reports revealed high overall quality of delivery (M=4.0, SD=0.86) and that caregivers were, on average, engaged across lessons (M=4.0, SD=0.4). Based on initial findings and facilitator input, the study team is considering additional reasons for non-participation by surveying those who did not attend after registering.

